# Factors Associated with Hip and Groin Pain in Elite Youth Football Players: A Cohort Study

**DOI:** 10.1186/s40798-021-00392-w

**Published:** 2021-12-19

**Authors:** Jacob Schoffl, Katherine Dooley, Peter Miller, Jess Miller, Suzanne J. Snodgrass

**Affiliations:** 1 School of Health Sciences, College of Health, Medicine and Wellbeing, The University of Newcastle, University Drive, Callaghan, NSW 2308 Australia; 2 School of Allied Health, Exercise and Sports Sciences, Charles Sturt University, Leeds Parade, Orange, NSW 2800 Australia; 3 NUmoves Physiotherapy, Ring Road, Callaghan, NSW 2308 Australia

**Keywords:** Groin pain, Soccer, Youth, Athletic injuries, Muscle injuries, Adductor, Muscle strength, Hip/pelvis/thigh

## Abstract

**Background:**

Despite hip and groin pain being commonly reported in elite youth football players, little evidence on risk factors exists. Risk factors in adult football players include reduced hip adductor strength and hip adductor/abductor strength ratios, and lower Copenhagen Hip and Groin Outcome Score (HAGOS) subscale scores. It is unknown if these factors are also predictive of pain development in youth football players.

**Objective:**

To identify whether preseason hip adductor and abductor strength and HAGOS subscale scores of male and female elite youth football players are associated with in-season or historical (lifetime) hip and groin pain.

**Methods:**

Preseason hip adductor and abductor strength testing and the HAGOS were undertaken by 105 elite male (*n* = 58) and female (*n* = 47) football players aged 11–15 years. Medical staff documented both players’ self-reported historical and in-season hip and groin pain. Univariate and multivariate logistic regression models were undertaken with main outcome measures in-season hip and groin pain and historical hip and groin pain and independent variables of hip muscle strength, hip muscle torque and HAGOS subscale scores.

**Results:**

Twenty-three players (21.9%) self-reported in-season hip and groin pain, while 19 players (18.1%) self-reported historical hip and groin pain. Pre-season hip adductor and abductor variables and HAGOS subscale scores failed to predict in-season hip and groin pain. However, a higher body mass index (odds ratio [OR] = 1.32; 95% CI 1.01, 1.73, *p* = .043) and being male (OR 5.71; 95% CI 1.65, 19.7) were associated with having in-season hip and groin pain (*R*^2^ = 0.211). There was also an association between historical hip and groin pain (*R*^2^ = 0.579) and both HAGOS subscale Quality of Life (odds ratio [OR] = 0.84; 95% CI 0.77, 0.91, *p* < .001) and mean abductor torque (OR = 11.85; 95% CI 1.52, 91.97; *p* = .018).

**Conclusion:**

Pre-season hip adductor and abductor strength and HAGOS subscale scores did not predict subsequent in-season hip and groin pain in elite youth football players. However, pre-season higher hip abductor strength and lower HAGOS scores were retrospectively associated with historical hip and groin pain.

## Key Points

- In the current study, pre-season hip adductor/abductor strength and HAGOS scores were unable to predict in-season hip and groin pain in elite youth football players.
- Increased pre-season body mass index in male elite youth football players is associated with subsequent hip and groin pain.
- Increased hip abductor torque and lower HAGOS Quality of Life subscale scores are associated with self-reported historical hip and groin pain.

## Background

The hip and groin is reported as the fourth most common injury location in elite youth football players [1], with recent pooled data reporting 7–33% of all time-loss injuries in elite youth football players occur in the ‘groin/adductor/pelvis/hips’ location [2]. Although not all hip and groin pain results in time-loss, as up to one-third of both adult and adolescent football players will continue to participate in games and training sessions despite the presence of groin pain [3, 4]. A recent cross-sectional study found 42% of elite youth football players self-reported groin pain symptoms in the previous season, irrespective of time-loss [5]. To prevent the development of hip and groin pain in this cohort risk factors that can be modified needs to be identified to allow for the development of preventative programs.

Currently the only prospective investigation in elite youth football players found those who experience early skeletal maturity were at a higher risk of groin strains than those who matured later [6]. As skeletal maturity is a non-modifiable risk factor, further investigations into modifiable risk factors are required. Modifiable risk factors for hip and groin pain in adult football players include increased weight, differences in external rotation range of motion between hips [7], higher levels of play, lower levels of sport specific training and reduced hip adductor strength [8]. As none of these modifiable risk factors for hip and groin pain have been prospectively investigated in elite youth football players, it is unclear if they are also predictive of pain in this population.

Reduced hip adductor strength has been identified as an intrinsic factor associated with hip and groin pain in adult male football players [9–12]. Hip adductor strength during pre-season testing is up to 5.4% weaker in players with previous-season groin pain than those without [11], while higher levels of pre-season hip adductor strength are protective against hip and groin pain [9]. Additionally, significantly reduced hip adductor-to-abductor strength ratios has been identified in adult football players, with a threshold of 80% hip adductor-to-abductor muscle strength ratio reported in players with groin problems [13, 14]. Despite in-season hip adductor strength and hip adductor-to-abductor strength ratio testing being employed as early detection tools for groin pain in elite youth football players [15], it is unclear if pre-season testing can be predictive of in-season groin pain.

The Copenhagen Hip And Groin Outcome Score (HAGOS) is a valid and reliable patient reported outcome measure for assessing hip and groin health in young to middle aged athletes [16] and is recommended for use in football cohorts [8]. The HAGOS has been shown to discriminate between football players with and without groin pain [17, 18], as well as identify players at risk of subsequent hip and groin pain [9, 15]. Lower preseason HAGOS subscale scores have been shown to be associated with an increased risk of hip and groin injuries in the subsequent season for elite male adult football players [9]. However, no identified study has investigated this relationship between HAGOS subscale scores and in-season hip and groin pain in a youth football population.

As most elite football players will commence training at a high level at an early age [19], recognising potential risk factors for hip and groin pain is important for developing training programs to reduce symptoms and sustain careers [20]. Therefore, the aims of this research were to identify whether pre-season hip adductor and abductor strength and HAGOS subscale scores of male and female elite youth football players are either (a) predictive of in-season historical hip and groin pain or (b) associated with historical hip and groin pain, irrespective of whether pain resulted in time-loss.

## Methods

### Study Design

A cohort study was conducted on elite football players (total *n* = 111; males *n* = 63; females *n* = 48). All players were recruited from a single club: Newcastle Jets A-League FC Academy in Newcastle, Australia. As all participants were under 18 years of age, written informed consent was obtained from both players and their parents/guardians. Ethics approval (protocol number: H-2018-0118) was provided by The University of Newcastle Human Research Ethics Committee. Pre-season data collection occurred in October 2018, consisting of the HAGOS questionnaire, reporting of historical (within their lifetime) hip and groin pain and hip adductor and abductor muscle strength testing. Subsequent in-season monitoring of all episodes of hip and groin pain, irrespective of time-loss, was collected during the ten-month playing season from November 2018 to August 2019.

### Participants

To define the population sample, the following participant characteristics were first collected: playing age group, gender, standing height (cm), body mass (kg) and dominant leg length from the lateral malleolus to the greater trochanter (cm). Participants were asked during injury history screening if they had ever experienced hip and groin pain in their lifetime. Hip and groin pain was defined as pain in the anterior hip, pubic region, inguinal regional and proximal adductor insertion (excluding lateral or posterior hip). In addition, participants were also asked to report their dominant limb: “which leg do you prefer to kick a ball with?”.

### Pre-season HAGOS Questionnaire

All participants completed the HAGOS, containing 37 questions answered on a Likert scale, grouped into six subscales: Pain, Symptoms, Activities of Daily Living (ADL), Sport & Recreational Activities (Sport/Rec), Participation in Physical Activity (PA) and Quality of Life (QOL) [16]. Each subscale was scored independently and then normalised to a 100-point scale, as per Thorborg et al. [16], with lower scores indicating greater hip and/or groin problems or disabilities. If a participant failed to answer more than one question for PA subscale or more than two questions for all other subscales (Symptoms; Pain; ADL; Sport/Rec; QOL), then the individual subscale was excluded from analysis and treated as ‘missing data’ [16].

### Pre-season Strength Testing

**Fig. 1:**
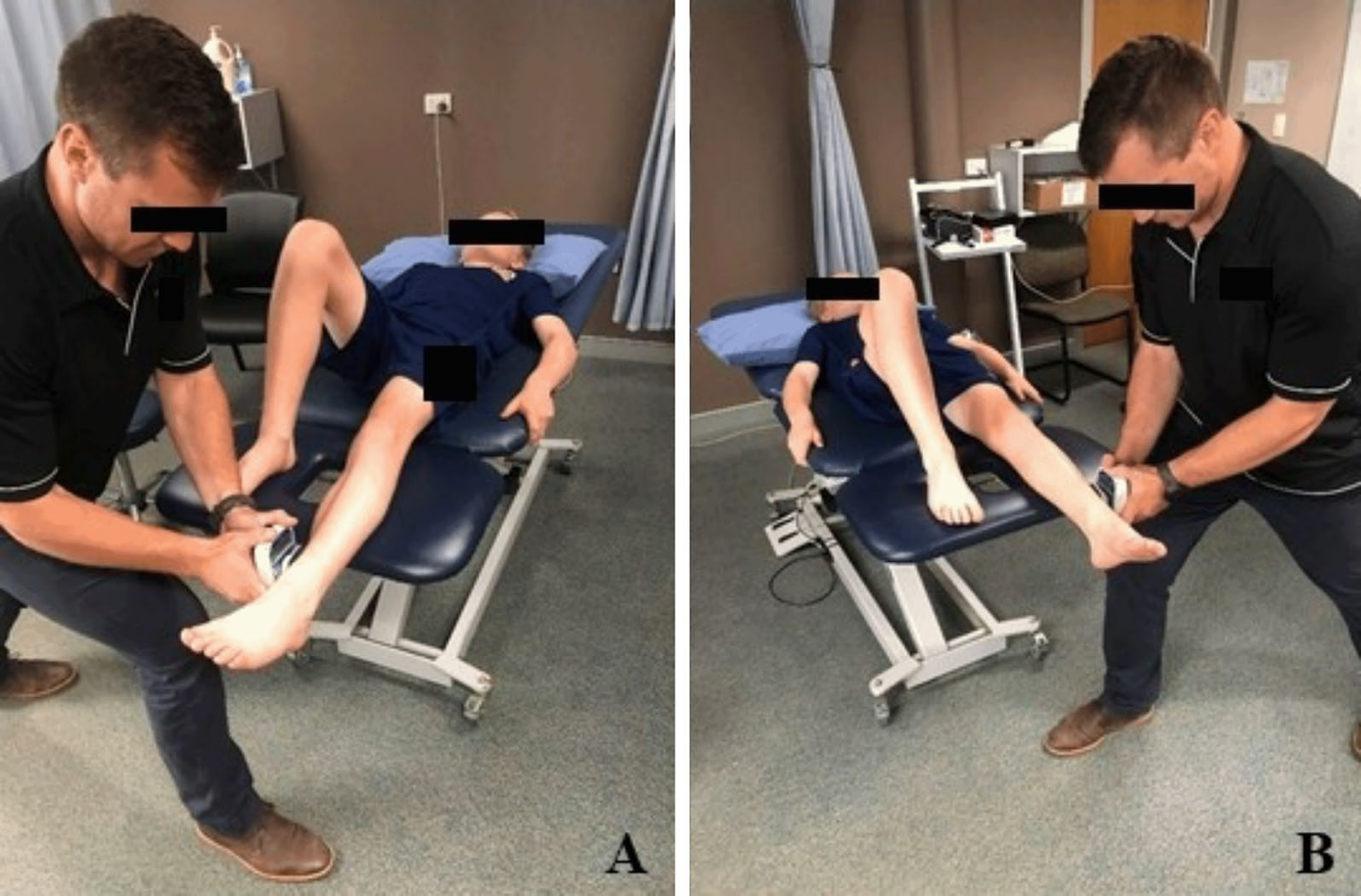
**A** Adductor and **B** abductor hip muscle strength testing positions. A hand-held dynamometer was used for testing and placed 5 cm proximal to either the **A** medial malleolus or **B** lateral malleolus. Players were allowed to stabilise themselves with their non-test leg and both hands holding onto plinth. Three break tests were performed, and the highest result was used (except when the highest value was > 10% higher than the next highest value, the second highest value was used)

Pre-season strength testing took place during a three-week period between playing seasons after squads had been selected and prior to the commencement of training. Unilateral hip adductor and abductor strength was recorded using a handheld dynamometer (HHD) (Lafayette model 01165 manual muscle tester with 7 cm × 3.5 cm pad). All strength tests were undertaken by five physiotherapists with a combined experience of 60 years (range 4–23 years), of whom two were titled musculoskeletal physiotherapists and a third had post-graduate qualifications. The strength testing procedures have previously been described and has been shown to have a high inter-rater intra-class coefficient between 0.86–0.93 for adductor and 0.87–0.98 for abductor strength testing [15, 21]. Participants lay supine with the leg being tested in 0° hip and knee extension and resistance applied 5 cm proximal to the malleolus, while the leg not being tested was in knee flexion with the foot on the plinth (Fig. 1). Participants completed a standardised warm-up of two five-second isometric repetitions against the HHD with ten second rests between repetitions prior to both hip adductor and abductor tests. The tests consisted of 3 ‘break’ repetitions, each held for ≤ 3 s with a 30 s rest period, for both the hip adductors and abductors. A ‘break’ test, defined as an eccentric muscle contraction, was used to determine participant’s maximal strength as it has been shown to be more sensitive in identifying male football players with adductor-related groin pain compared to isometric testing [13]. Examiners instructed participants to “push as hard as you can” and repeated the encouragement “keep going”’ three times for each test. For each test participants were allowed to stabilise themselves by gripping the side of the plinth with their hands and pressing the non-testing leg against the plinth [15, 21].

To attain a reproducible result that represented an individual’s maximal effort, the highest value for the three testing repetitions was used for analysis, unless the highest value was > 10% of the player’s other two values. If this occurred, the highest value was considered an outlier and the second highest of the three values was taken [22]. If a testing repetition was painful, the test was stopped, and this value was excluded from analyses. Hip adductor and abductor strength was measured in Newtons (N), with muscle torque calculated by multiplying the highest accepted strength value by leg length in metres (measured from the lateral malleolus to the greater trochanter of the player’s dominant leg) and then divided by body mass in kilograms [13]. Leg length measurements were taken of the dominant leg only due to time constraints and the limited accuracy of tape measures in detecting subtle between-limb differences. Eccentric hip adductor-to-abductor strength ratio was calculated for each leg [15]. Between limb difference was calculated by subtracting the dominant side from the non-dominant side for force values (N) and torque (N/Kg).

### In-Season Monitoring

Players were advised to report any pain or injuries to medical staff at both games and training sessions, irrespective of whether treatment was required. This resulted in both time-loss and non-time-loss hip and groin pain being recorded on a weekly basis [4]. If hip and groin pain was reported by a player, it was then assessed at the first training session of the weekly schedule by a registered physiotherapist with 20 years of musculoskeletal physiotherapy experience. Players with hip and groin pain were identified by the physiotherapist from electronic records kept by physiotherapists and trainers on game days, technical director reports of injuries from coach’s post game reports, players who were not training at this session, and players who presented to the physiotherapist with pain at the training session or the clinic. The physiotherapist assessed all of these identified players at the first weekly training session, with these data entered into an excel spreadsheet that became the final data record used for analysis. Assessment of the hip and groin region included but was not limited to functional movements (e.g. squatting, jumping, kicking football), passive and active joint range of motion, manual muscle strength testing and combined hip-joint movement tests (flexion adduction internal rotation and flexion adduction external rotation). The physiotherapist determined a clinical diagnosis based on their assessment.

### Statistical Analysis

Hip and groin pain prevalence in youth elite players was calculated for both in-season hip and groin pain and historical hip and groin pain by dividing the number of players reporting pain by the total number of players included in the study. For analysis, players were categorised as either in-season hip/groin pain (at least one episode) or no in-season hip and groin pain. Descriptive statistics were calculated for each variable with male and female data stratified, as both strength [23] and the experience of pain [24] differs between sexes. For the analysis of strength variables both legs of an individual were averaged as there were no differences between painful and non-painful limbs in players with pain, or between left and right limbs of asymptomatic players, or their left and right as compared to the left and right of pain players matched by weight and height. Data normality was assessed through Shapiro–Wilk tests and visualisation of histograms. Differences between pain and asymptomatic players were calculated using independent t-tests for parametric data or Mann–Whitney-U tests for non-parametric data.

Logistic regression models determined factors associated with having in-season hip and groin pain. Prior to logistic regression, Pearson’s coefficients were used to examine potential correlations between independent variables, with all strength variables found to be correlated, and the majority of HAGOS subscales correlated. Due to the large number of possible candidate variables, univariate modelling was used to determine which variables were to be included in the multivariate model. Variables that underwent univariate modelling were leg length, body mass index (BMI), gender, playing age group at time of testing (ages 11, 12, 13 or 14 years) historical hip and groin pain, hip abductor muscle torque, hip adductor muscle torque, hip adductor/abductor strength ratio and HAGOS subscales Pain, Symptoms, ADL, Sport/Rec and QOL. Variables with *p* < 0.25 in the univariate models were then included in a multi-variate model analysed using the backwards (Wald) method [25]. A model combining both male and female sexes was used as male and female multi-variate models had similar results to the combined model. The same methodological process was also used for separate analyses that examined differences in hip muscle strength and HAGOS subscale scores for players with either historical hip and groin pain or no historical hip and groin pain. Where variables representing a construct (i.e. strength, HAGOS subscales) were correlated, selection of variables for the multivariate model was based on lowest p-value and/or highest Nagelkerke *R*^2^ from the univariate models. SPSS v 27.0 (IBM SPSS Statistics, IBM Corp., Armonk, NY) was used for analyses.

## Results

### Participants

Of the 111 players, 106 players consented to participate in this study (male *n* = 59; females *n* = 47). One male was excluded as no strength data were recorded, resulting in 105 players included in analysis (cohort mean age 12.7 ± 1.0 years, males 12.5 ± 1.1, females 12.9 ± 0.8). Due to missing leg length data for another male, torque values were unable to be calculated for this player. During pre-season hip muscle strength testing 6 players experienced pain (3 males, 1 with pain during abductor testing, 2 with pain during both adductor and abductor testing; 3 females with pain during abductor testing) with three of these players (2 males, 1 female) reporting subsequent in-season hip and groin pain.

### Hip and Groin Pain Prevalence

For all participants included in analysis, 23 (21.9%) players self-reported at least one incidence of either time-loss or non-time-loss in-season hip and groin pain (18 male, 5 female). Eighteen (17.1%) players experienced time-loss groin pain (14 male, 4 female), while 5 (4.8%) players experienced non-time-loss groin pain (4 male, 1 female). Of the 23 players who reported hip and groin pain within the season, 30.4% experienced more than one incidence (7 male, 0 female), resulting in a total of 30 episodes of hip and groin pain. The prevalence of self-reported historical hip and groin pain within a players’ lifetime was reported in 18.1% of the 105 total players at the time of pre-season data collection (13 male, 6 female). All players self-reporting historical hip and groin pain also experienced at least one incidence of in-season hip and groin. The physiotherapist’s clinical diagnosis for the in-season hip and groin injuries determined that five players had adductor-related injuries (4 male, 2 female), 11 players had psoas-related injuries (9 male, 2 female), and seven were mixed presentations or non-specific (5 male, 2 female).

### Preseason Measures Associated with Hip and Groin Pain

Descriptive statistics and between-groups differences for both in-season hip and groin pain vs. no in-season hip and groin pain and historical hip and groin pain vs. no historical hip and groin pain players, stratified by sex, are reported in Tables 1, 2 and 3. Male players who developed in-season pain had a significantly higher BMI than players who did not (Table 1), while female players who developed in-season pain had lower between limb differences for both hip adductor strength and torque than those with no pain (*p* < 0.05; Table 2). Both leg length (Table 1) and hip abductor values (strength and torque) (Table 2) were significantly increased during pre-season testing in all players with historical hip and groin pain compared to those with no historical pain (*p *< 0.05). For female players, between limb difference for both hip abductor strength and torque were higher in those with historical hip and groin pain vs. no historical pain (*p* < 0.01). Median preseason HAGOS subscale scores (Table 3) were only lower for players who reported historical hip and groin pain for all six subscales.

**Table 1:** Mean (SD) baseline characteristics of elite youth football players (aged 11–15), divided into in-season hip and groin pain and no in-season hip and groin pain, and historical hip and groin pain and no historical hip and groin pain, stratified by sex

Characteristic	All	In-season hip and groin pain		Difference between groups	*p*	Historical hip and groin pain		Difference between groups	*p*
	All (*n* = 105) Male (*n* = 58) Female (*n* = 47)	Pain All (*n* = 23) Male (*n* = 18) Female (*n* = 5)	No pain All (*n* = 82) Male (*n* = 40) Female (*n* = 42)	95% CI		Pain All (*n* = 19) Male (*n* = 13) Female (*n* = 6)	No pain All (*n* = 86) Male (*n* = 45) Female (*n* = 41)	95% CI	
Weight (kg)	46.8 (9.1)	49.2 (10.4)	46.2 (8.7)	3.1 (− 1.2–7.3)	.156	49.7 (8.7)	46.2 (9.1)	3.5 (− 1.1–8.1)	.131
Male	45.9 (9.9)	48.9 (11.7)	44.5 (8.8)	4.4 (− 1.1–10.0)	.117	48.5 (9.0)	45.1 (10.1)	3.4 (− 2.8–9.6)	.277
Female	48.0 (8.0)	50.2 (3.4)	47.7 (8.4)	2.5 (− 5.2–10.2)	.515	52.2 (8.1)	47.4 (7.9)	4.8 (− 2.2–11.8)	.172
Standing height (cm)	156.8 (10.1)	158.7 (11.7)	156.2 (9.6)	2.4 (− 2.3–7.1)	.308	160.7 (10.3)	155.9 (9.8)	4.8 (− 0.2–9.8)	.060
Male	156.8 (12.1)	158.5 (13.1)	156.1 (11.7)	2.4 (− 4.6–9.3)	.498	160.8 (12.1)	155.7 (12.0)	5.1 (− 2.5–12.7)	.180
Female	156.6 (6.8)	159.3 (4.8)	156.3 (7.0)	3.0 (− 3.5–9.5)	.363	160.3 (5.6)	156.1 (6.9)	4.2 (− 1.7–10.2)	.159
Leg length (cm)^a^	73.7 (6.1)	75.4 (6.9)	73.3 (5.8)	2.1 (− 0.8–5.0)	.148	76.5 (6.1)	73.1 (5.9)	3.4 (0.3–6.4)	.032
Male	73.4 (7.0)	75.1 (7.8)	72.7 (6.6)	2.4 (− 1.7–6.4)	.244	76.1 (7.1)	72.7 (6.8)	3.4 (− 1.1–7.8)	.138
Female	74.1 (4.8)	76.5 (3.4)	73.9 (4.9)	2.6 (− 2.0–7.1)	.257	77.3 (3.9)	73.6 (4.8)	3.7 (− 0.4–7.8)	.075
Body Mass Index (kg/m^2^)	18.9 (2.1)	19.3 (1.8)	18.8 (2.1)	0.6 (− 0.4–1.5)	.249	19.1 (2.0)	18.8 (2.1)	0.3 (− 0.7–1.3)	.557
Male	18.4 (1.7)	19.2 (2.0)	18.1 (1.4)	1.1 (0.2–2.0)	.018	18.6 (1.4)	18.4 (1.8)	0.2 (− 0.8–1.3)	.651
Female	19.4 (2.3)	19.8 (1.2)	19.4 (2.5)	0.4 (− 1.9–2.7)	.723	20.3 (2.7)	19.3 (2.3)	0.9 (− 1.1–3.0)	.366

^a^1 Male participant had no leg length value recorded; data for leg length include all (n = 104); pain (n = 22)

**Table 2:** Mean (SD) for hip adductor and abductor muscle strength, torque and ratios for youth footballers (aged 11–15), divided into in-season hip and groin pain and no in-season hip and groin pain, and historical hip and groin pain and no historical hip and groin pain, stratified by sex

Characteristic	All	In-season hip and groin pain		Difference between groups	p	Historical hip and groin pain		Difference between groups	p
	All (*n* = 105) Male (*n* = 58) Female (*n* = 47)	Pain All (*n* = 23) Male (*n* = 18) Female (*n* = 5)	No pain All (*n* = 82) Male (*n* = 40) Female (*n* = 42)	Pain minus no pain (95% CI)		Pain All (*n* = 19) Male (*n* = 13) Female (*n* = 6)	No pain All (*n* = 86) Male (*n* = 45) Female (*n* = 41)	Historical pain minus no pain (95% CI)	
Adductors (N)^a^	13.5 (3.7)	13.7 (3.6)	13.4 (3.8)	0.3 (− 1.4 to 2.1)	.728	14.7 (3.9)	13.2 (3.7)	1.5 (− 0.3–3.4)	.110
Male	14.3 (4.2)	13.9 (3.9)	14.5 (4.3)	− 0.5 (− 2.9 to 1.9)	.669	14.9 (4.3)	14.1 (4.2)	0.8 (− 1.8 to 3.5)	.547
Female	12.5 (2.8)	13.0 (2.9)	12.4 (2.9)	0.5 (− 2.2 to 3.3)	.694	14.3 (3.1)	12.2 (2.7)	2.1 (− 0.3 to 4.5)	.091
Abductors (N)	12.4 (3.1)	12.8 (3.2)	12.2 (3.1)	0.6 (− 0.9 to 2.0)	.417	14.0 (2.9)	12.0 (3.0)	2.0 (0.5–3.5)	.008
Male	12.7 (3.1)	12.7 (3.3)	12.7 (3.1)	0.0 (− 1.8 to 1.8)	.996	13.9 (3.3)	12.4 (3.0)	1.6 (− 0.4 to 3.5)	.110
Female	11.9 (3.0)	13.2 (3.2)	11.8 (3.0)	1.4 (− 1.5 to 4.3)	.329	14.2 (2.0)	11.6 (3.0)	2.7 (0.1–5.2)	.042
Adductor torque (N/Kg)^b^	2.1 (0.5)	2.1 (0.5)	2.1 (0.5)	0.0 (− 0.2 to 0.2)	.950	2.3 (0.5)	2.1 (0.5)	0.2 (− 0.1 to 0.4)	.119
Male	2.3 (0.5)	2.1 (0.5)	2.3 (0.5)	− 0.2 (− 0.5 to 0.1)	.228	2.3 (0.5)	2.2 (0.5)	0.1 (− 0.2 to 0.4)	.542
Female	1.9 (0.4)	2.0 (0.5)	1.9 (0.4)	0.0 (− 0.3 to 0.4)	.786	2.1 (0.5)	1.9 (0.4)	0.2 (− 0.1 to 0.6)	.172
Abductor torque (N/Kg)	1.9 (0.4)	2.0 (0.4)	1.9 (0.4)	0.1 (− 0.1 to 0.2)	.534	2.2 (0.4)	1.9 (0.4)	0.3 (0.1–0.5)	.002
Male	2.0 (0.4)	2.0 (0.4)	2.0 (0.4)	− 0.1 (− 0.3 to 0.1)	.503	2.2 (0.4)	2.0 (0.3)	0.3 (0.0–0.5)	.026
Female	1.8 (0.4)	2.0 (0.5)	1.8 (0.4)	0.2 (− 0.2 to 0.5)	.312	2.1 (0.3)	1.8 (0.4)	0.3 (0.0–0.6)	.054
Adductor/abductor strength ratio (N)^c^	1.1 (0.2)	1.1 (0.2)	1.1 (0.2)	0.0 (− 0.1 to 0.1)	.538	1.1 (0.2)	1.1 (0.2)	− 0.1 (− 0.1 to 0.0)	.166
Male	1.1 (0.2)	1.1 (0.2)	1.1 (0.2)	0.0 (− 0.1 to 0.1)	.498	1.1 (0.2)	1.1 (0.2)	− 0.1 (− 0.2 to 0.0)	.205
Female	1.1 (0.1)	1.0 (0.1)	1.1 (0.1)	− 0.1 (− 0.2 to 0.1)	.278	1.0 (0.2)	1.1 (0.1)	− 0.1 (− 0.2 to 0.1)	.292
Between limb difference adductors (N)^d^	0.494 (2.1)	0.457 (2.7)	0.505 (2.0)	0.0 (− 1.1 to 1.0)	.924	0.5 (3.5)	0.5 (1.7)	0.0 (− 1.0 to 1.1)	.943
Male	0.686 (2.2)	1.1 (1.7)	0.5 (2.3)	0.6 (− 0.6 to 1.9)	.308	0.9 (2.8)	0.6 (2.0)	0.3 (− 1.1 to 1.7)	.679
Female	0.3 (2.1)	− 1.9 (4.2)	0.5 (1.6)	− 2.5 (− 4.3 to − 0.6)	.012	− 0.3 (5.0)	0.3 (1.4)	− 0.6 (− 2.5 to 1.2)	.492
Between limb difference abductors (N)	− 0.412 (2.2)	− 0.630 (1.97)	− 0.351 (2.3)	− 0.3 (− 1.3 to 0.8)	.592	0.3 (3.4)	− 0.6 (1.8)	0.9 (− 0.2 to 2.0)	.107
Male	− 0.791 (2.2)	− 0.694 (2.2)	− 0.835 (2.3)	0.1 (− 1.1 to 1.4)	.827	− 0.5 (3.4)	− 0.9 (1.8)	0.3 (− 1.1 to 1.7)	.658
Female	0.1 (2.1)	− 0.4 (1.0)	0.1 (2.1)	− 0.6 (− 2.5 to 1.5)	.606	2.2 (2.9)	− 0.3 (1.7)	2.5 (0.8–4.1)	.005
Between limb difference adductor torque (N/Kg)	0.08 (0.3)	0.05 (0.4)	0.08 (0.3)	0.0 (− 0.2–0.1)	.693	0.1 (0.5)	0.1 (0.3)	0.0 (− 0.2 to 0.2)	.820
Male	0.1 (0.3)	0.1 (0.2)	0.1 (0.4)	0.1 (− 0.1–0.3)	.517	0.1 (0.5)	0.1 (0.3)	0.0 (− 0.2 to 0.2)	.935
Female	0.0 (0.3)	− 0.3 (0.6)	0.1 (0.2)	− 0.4 (− 0.6 to − 0.1)	.011	0.0 (0.7)	0.1 (0.2)	− 0.1 (− 0.4 to 0.2)	.479
Between limb difference abductor torque (N/Kg)	− 0.07 (0.3)	− 0.08 (0.3)	− 0.06 (0.4)	0.0 (− 0.2 to 0.2)	.865	0.0 (0.6)	− 0.1 (0.3)	0.1 (− 0.2 to 0.4)	.131
Male	− 0.1 (0.4)	− 0.1 (0.3)	− 0.1 (0.4)	0.1 (− 0.2 to 0.3)	.608	− 0.1 (0.6)	− 0.1 (0.3)	0.0 (− 0.2 to 0.3)	.813
Female	0.0 (0.3)	− 0.1 (0.2)	0.0 (0.3)	− 0.1 (− 0.4 to 0.2)	.635	0.3 (0.4)	− 0.1 (0.3)	0.4 (0.1–0.6)	.003

^a^Strength: the highest of 3 attempts of a break test was used (except when the highest value was > 10% more than the next highest value, the 2nd highest value was used)

^b^Torque calculated using the formula: Strength value of muscle group × leg length in meters/weight in kilograms

^c^Adductor/abductor calculated by averaging the muscle groups for both sides then dividing the strength of the adductors by the strength of the abductors

^d^Between limb difference calculated by subtracting the non-dominant leg muscle group from the dominant leg muscle group

**Table 3:** Median (IQR, 25th–75th percentiles) for each subscale of the Copenhagen Hip And Groin Outcome Score (HAGOS) for youth footballers (aged 11–15), divided into in-season hip and groin pain and no in-season hip and groin pain, and historical hip and groin pain and no historical hip and groin pain, stratified by sex

HAGOS subscale^a^	All	In-season hip and groin pain		*p*^†^	Historical hip and groin pain		*p*^†^
		Pain	No pain		Historical pain	No pain	
	All (*n* = 105) Male (*n* = 58) Female (*n* = 47)	All (*n* = 23) Male (*n* = 18) Female (*n* = 5)	All (*n* = 82) Male (*n* = 40) Female (*n* = 42)		All (*n* = 19) Male (*n* = 13) Female (*n* = 6)	All (*n* = 86) Male (*n* = 45) Female (*n* = 41)	
Pain	100.0 (98.0–100.0)	100.0 (95.0–100.0)	100.0 (98.0–100.0)	.098	95.0 (90.0–100.0)	100.0 (100.0)	< .001
Male	100.0 (98.0–100.0)	100.0 (90.0–100.0)	100.0 (98.0–100.0)	.230	95.0 (90.0–100.0)	100.0 (100.0)	.010
Female	100.0 (98.0–100.0)	98.0 (95.0–100.0)	100.0 (98.0–100.0)	.219	95.0 (92.3–98.5)	100.0 (98.0–100.0)	.002
Symptoms	96.0 (89.0–100.0)	93.0 (82.0–100.0)	96.0 (89.0–100.0)	.110	89.0 (75.0–100.0)	96.0 (93.0–100.0)	.019
Male	96.0 (89.0–100.0)	94.5 (81.3–100.0)	96.0 (89.0–100.0)	.274	89.0 (77.0–98.0)	96.0 (91.0–100.0)	.060
Female	96.0 (89.0–100.0)	93.0 (84.0–96.5)	96.0 (92.0–100.0)	.219	89.5 (75.0–100.0)	96.0 (93.0–100.0)	.187
Activities of daily living	100.0 (100.0)	100.0 (100.0)	100.0 (100.0)	.917	100.0 (95.0–100.0)	100.0 (100.0)	< .001
Male	100.0 (100.0)	100.0 (100.0)	100.0 (100.0)	.849	100.0 (90.0–100.0)	100.0 (100.0)	< .001
Female	100.0 (100.0)	100.0 (100.0)	100.0 (100.0)	.541	100.0 (95.0–100.0)	100.0 (100.0)	.004
Sport and recreational activities	100.0 (97.0–100.0)	100.0 (94.0–100.0)	100.0 (97.0–100.0)	.176	91.0 (84.0–100.0)	100.0 (100.0)	< .001
Male	100.0 (97.0–100.0)	100.0 (91.5–100.0)	100.0 (97.0–100.0)	.222	84.0 (81.0–100.0)	100.0 (98.5–100.0)	< .001
Female	100.0 (100.0)	100.0 (98.5–100.0)	100.0 (100.0)	.828	100.0 (86.3–100.0)	100.0 (100.0)	0.285
Participation in physical activities	100.0 (100.0)	100.0 (100.0)	100.0 (100.0)	.147	100.0 (88.0–100.0)	100.0 (100.0)	< .001
Male	100.0 (100.0)	100.0 (100.0)	100.0 (100.0)	.400	100.0 (69.0–100.0)	100.0 (100.0)	< .001
Female	100.0 (100.0)	100.0 (56.5–100.0)	100.0 (100.0)	.166	94.0 (69.3–100.0)	100.0 (100.0)	< .001
Quality of life	100.0 (90.0–100.0)	100.0 (80.0–100.0)	100.0 (95.0–100.0)	.080	80.0 (65.0–90.0)	100.0 (100.0)	< .001
Male	100.0 (95.0–100.0)	100.0 (80.0–100.0)	100.0 (100.0)	.061	80.0 (70.0–95.0)	100.0 (100.0)	< .001
Female	100.0 (90.0–100.0)	100.0 (72.5–100.0)	100.0 (90.0–100.0)	.507	80.0 (73.8–88.8)	100.0 (92.5–100.0)	.001

^†^*P* value calculated using Mann–Whitney U

^a^Each subscale scored out of 100, with lower scores indicating worse problems (i.e. a score of 100 is no problems)

From the univariate models for in-season hip and groin pain, the following variables met the criterion for inclusion in the multivariate modelling: gender, BMI, leg length, and HAGOS subscales for Pain, Symptoms, Sport/Rec, PA and QOL. In the final multivariate model for in-season hip and groin pain, higher BMI (odds ratio [OR] 1.32; 95% CI 1.01, 1.73) and male gender (OR 5.71; 95% CI 1.65, 19.7) were associated with developing in-season hip and groin pain when accounting for HAGOS Symptom subscale score, explaining 21% of the variance (Table 4). Other variables (hip abductor torque, hip adductor torque, hip adductor/abductor strength ratio, leg length, playing age group, historical hip and groin pain, other HAGOS subscale scores) were not significantly associated with in-season hip and groin pain. For historical hip and groin pain, variables that met the criteria for inclusion in multivariate modelling were gender, leg length, abductor torque, adductor torque, adductor/abductor strength ratio, and all HAGOS subscales. In the final multivariate model, lower HAGOS QOL subscale scores (OR 0.84; 95% CI 0.77, 0.91) and greater abductor torque (OR 11.85; 95% CI 1.53, 91.97) were associated with historical hip and groin pain, explaining 58% of the variance. No other variables were significantly associated with historical hip and groin pain.

**Table 4:** Final logistic regression models (backwards Wald) indicating (1) pre-season variables that predict experiencing 1 or more episodes of in-season hip and groin pain, and (2) variables that were associated with historical hip and groin pain in male and female youth football players (aged 11–15)

	Odds ratio	95% CI	*p* value
*Predictors for players who experience 1 or more episodes of hip/groin pain during the season (R*^*2*^ = *0.211)*			
Body mass index (kg/m^2^)	1.32	1.01–1.73	.043
Gender (male)	5.71	1.65–19.7	.006
Symptoms*	0.95	0.89–1.00	.065
*Variables associated with having a history of hip/groin pain (R*^*2*^ = *0.579)*			
Quality of life^a^	0.84	0.77–0.91	< .001
Abductor torque (N/Kg)	11.85	1.53–91.97	.018

^a^HAGOS subscale scored out of 100, with lower scores indicating worse problems (i.e. a score of 100 is no problems)

## Discussion

The aim of the current study was to determine if hip adductor and abductor muscle strength and HAGOS subscale scores can be used to predict which players may experience hip and groin pain during the season, and whether they are associated with historical hip and groin pain in elite youth football players (age 11–15 years). Preseason hip muscle strength and HAGOS subscale scores were found not to be associated with nor could they predict in-season hip and groin pain in this youth population. Instead, multivariate logistic regression identified an association between in-season hip and groin pain and the variables of a higher BMI and male gender. Males had a higher proportion of in-season and historical hip and groin pain compared to females which is consistent with previous studies [4, 26]. Greater abductor torque and lower HAGOS QOL subscale scores were associated with historical hip and groin pain.

A higher BMI was predictive of in-season pain (Table 4), and mean leg length was longer for players for pain compared to those who had not experienced pain (Table 1). These findings suggest that larger, and potentially more physically mature players were more commonly reporting hip and groin pain. This is not the first time more physically mature elite youth football players have been associated with hip and groin pain, as skeletal maturity analysed via radiological examination has been shown to be a risk for groin strains [6]. However in contrast, the current study also found no differences in values between players of different playing age groups suggesting maturation status alone is not effective for predicting in-season hip and groin pain elite youth football players. More recent research supports this conclusion. When calculating maturation status via chronological age, standing height, body mass and mid-parental height 71% of injuries experienced by male elite youth football players occurred in those whose maturation status was “on time” [1]. The findings of previous research and the current study suggest that, dependent on how biological maturity is measured, it may be a non-modifiable risk factor for hip and groin pain in elite youth football players.

No relationship was found in the current study between pre-season hip muscle strength and in-season hip and groin pain of elite youth male and female football players aged 11–15. This contrasts with research in adult male football players that has shown reduced bilateral hip adductor strength in supine is associated with the occurrence of in-season groin [9, 11]. Although differences in results may be attributed to the types of muscle contractions, number of repetitions and length of time of muscle contraction undertaken in other studies, the age of the players may also be a contributing factor as the current study consisted of younger players, aged between 11 and 15 years of age. During these ages, players are undergoing considerable growth with peak growth rate estimated to occur on average at 13.5 years for males and 11.5 years for females [27]. This suggests recent evidence indicating hip adductor and abductor strength remains consistent within a single season found in professional adult male football players [28] should not be applied to adolescent players. In adolescent players many risk factors appear to be at play, such as maturation rate and possible (pre)pubertal hormonal changes [6, 29], historical hip and groin injury, level of play and sport specific training [26], which highlights the complexity of injury prevention. Regular in-season monitoring and testing of eccentric hip adductor strength has previously been suggested as an effective early detection and management strategy of hip and groin pain in elite football players < 16 years [15]. It is suggested this method be employed in male players, particularly those with higher BMIs, who were identified in the current study to be at greater risk of developing in-season hip and groin pain.

Preseason HAGOS subscale scores were not found to be predictive of in-season hip and groin pain in adolescent elite football players. These results differ to a previous study by Bourne et al. [9] in football players aged 24.5 ± 5.1 who found that higher HAGOS scores were associated with reduced risk of subsequent hip and groin pain The HAGOS is designed to capture the ongoing functional deficits of hip and groin pain and therefore is not expected to be effective as a prediction tool for hip and groin pain. In the current study, players with historical hip and groin pain scored significantly lower on all six of the HAGOS subscales. These findings are consistent with previous studies in adult football players following hip and groin pain [17, 30] and suggests hip and groin pain episodes can lead to ongoing problems in youth football players. From these findings, it appears pre-season HAGOS values are an ineffective measure in predicting in-season hip and groin pain but may be a useful tool for clinicians to quantify the ongoing functional impacts associated with hip and groin pain in youth football players.

In the current study, historical hip and groin pain was not associated with in-season hip and groin pain, irrespective of time-loss. This differs to the conclusions of a 2015 systematic review identifying previous hip and groin pain to be a risk factor for hip and groin pain in adult sporting populations [26]. Two possible reasons exist for this discrepancy: (1) players in the current study were aged 11–15 and likely had fewer years of playing at a competitive level, resulting in less injury risk exposure and potential development of hip and groin pain; and (2) the current study pooled time-loss and non-time loss hip and groin pain when defining both historical and in-season pain. Recent research on adult male football players found no association between time-loss historical and subsequent time-loss hip and groin pain, whereas time-loss historical hip and groin pain was associated with ‘severe’ hip and groin symptoms, as measured by the HAGOS [7]. This suggests that how hip and groin pain is defined can impact on whether an association can be detected between historical and in-season hip and groin pain. Due to the small number of players with non-time-loss hip and groin pain (*n* = 5), analysis of separate analysis of this group was not able to be conducted.

There were several limitations to the current study. HHD may not be sensitive enough to detect strength differences within this cohort [31]. A potential selection bias may have reduced the generalisability of this study as participants were recruited from one elite youth football club. Irrespective of this, the included players are a good representation of elite youth football players aged 11–15 as they train four times a week and compete in the highest level of youth football competition in Australia. Strength and HAGOS data were recorded at one time point, which is consistent with other prospective cohort studies in sporting populations [32, 33], but may fail to account for potential changes in hip strength over a season previously seen in players over 16 years [9, 34]. Potential under-reporting of within season pain was possible as players may not want to be perceived as having a problem [35]. However, as both players and coaching staff was encouraged to report players’ pain it is plausible all occurrences of pain were captured or were below a clinical threshold. Additionally, the reporting of historical hip and groin pain during any time in a players’ life may have resulted in recall bias and either the under- or over-estimation of historical hip and groin pain. Football players’ hip and groin pain was not clinically classified according to the Doha agreement [8]. The reliability of the HAGOS questionnaire has not been previously reported in populations younger than 18 years of age; however, it has previously been used for in-season monitoring of elite youth football players [15]. The HAGOS was able to identify players with hip and groin problems in both the aforementioned study [15] and the current study, suggesting it may be useful in younger sporting populations. Future research should investigate its reliability and validity in athletes < 18 years of age with reference values developed for this population.

## Conclusion

This is the first study to prospectively investigate the association between hip adductor and abductor strength and HAGOS subscale scores in male and female elite football players aged 11–15 years. Although pre-season hip adductor and abductor muscle strength and HAGOS values were not associated with in-season hip and groin pain, male players with a higher BMI were at greater risk of developing in-season hip and groin pain. As pre-season strength testing did not predict injury, regular in-season monitoring might be considered to determine if it may assist early detection of hip and groin injuries which has potential to reduce the severity of symptoms in this cohort. Historical hip and groin pain was not associated with in-season hip and groin pain, but those with a history had ongoing hip and groin problems as quantified using the HAGOS. Historical hip and groin pain was found to be strongly associated with lower HAGOS Quality of Life subscale scores and higher hip abductor torque values, suggesting hip and groin pain episodes can lead to ongoing problems in youth football players.

## Data Availability

The datasets used and/or analysed during the current study are available from the corresponding author on reasonable request.

## Abbreviations

ADL: Copenhagen Hip and Groin Outcome Score subscale function in daily living
BMI: Body Mass Index
HAGOS: The Copenhagen Hip and Groin Score
HHD: Handheld dynamometer
N: Newtons
N/Kg: Newtons per kilogram
QOL: Copenhagen Hip and Groin Outcome Score subscale hip and/or groin quality of life
PA: Copenhagen Hip and Groin Outcome Score participation in physical activities
Sport/Rec: Copenhagen Hip and Groin Outcome Score subscale function in sport and recreation
